# Novel protective retrieval bag for safe removal of gastric gastrointestinal stromal tumor after endoscopic full-thickness resection

**DOI:** 10.1055/a-2072-3959

**Published:** 2023-05-10

**Authors:** Miguel Fraile-López, Noriya Uedo, Yoji Takeuchi, Tomoki Michida

**Affiliations:** 1 Gastroenterology and Hepatology Department, Clinical and Translational Research in Digestive Diseases, Valdecilla Research Institute (IDIVAL), Marqués de Valdecilla University Hospital, Santander, Spain; 2 Department of Gastrointestinal Oncology, Osaka International Cancer Institute, Osaka, Japan

Endoscopic resection of gastric subepithelial lesions (GSELs) is gaining in popularity owing to its good results and less invasive nature compared to surgery 1 2 . However, extracting specimens through the gastroesophageal junction or upper esophageal sphincter can be technically challenging and may cause fragmentation or capsule damage, which may lead to an inaccurate histopathological evaluation 2 .

Here, we report of a new retrieval device (ENDO CARRY, Hakko Co., Ltd., Nagano, Japan) with a drawstring-type plastic bag 3 that facilitates the safe retrieval of resected GSELs. This device consists of a larger transparent plastic bag (60 mm) that is not pulled into the sheath when it is completely closed, which prevents specimen damage during the extraction ( Fig. 1 ). This device cannot be used through the working channel, so for its insertion the transparent bag is placed on the tip of the endoscope, which allows reaching the stomach while seeing through the bag. For specimen retrieval, the bag is opened into the stomach and the GSEL is introduced inside using a grasping forceps or using the latter to directly trap the GSEL with the bag ( Video 1 ).

**Fig. 1:**
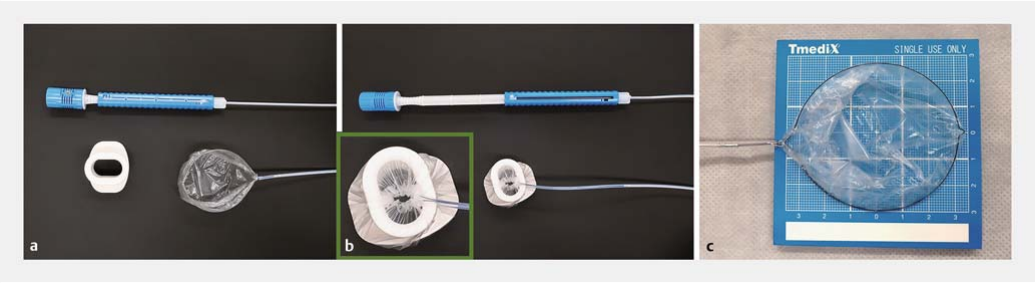
Modified ENDO CARRY bag for protective retrieval of gastric subepithelial lesions. **a** Retrieval device in an open position. **b** Retrieval device in the closed position with the bag out of the sheath. **c** Major (80 mm) and minor (60 mm) diameter with complete open position.

**Video 1** Protective retrieval bag for safe removal of gastric gastrointestinal stromal tumor after endoscopic full-thickness resection.


Our experience consists of nine GSEL retrievals by using this bag. The median GSEL size was 22 × 26 mm [19–25, 23–38]; four (44 %) were grasped with forceps into the bag, and five (56 %) were directly grasped preventing pseudocapsule injury in all of them ( Fig. 2 ).

**Fig. 2:**
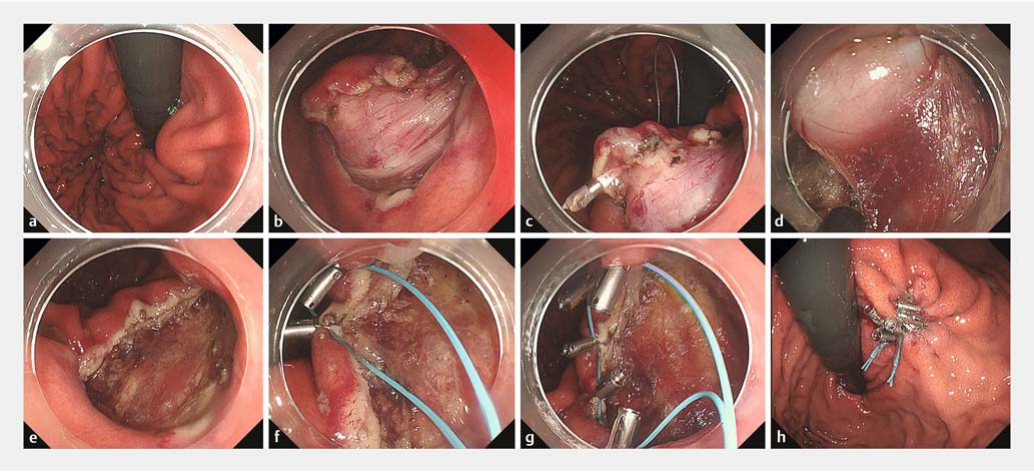
Endoscopic full-thickness resection of a gastric gastrointestinal stromal tumor (GIST) with an endoluminal component. **a** Endoscopic retroflex view with subcardiac/lesser curvature GIST. **b** Submucosal dissection completed with complete tumor exposure. **c** Clip-line traction method applied before muscle cut. **d** Full-thickness resection almost completed. **e** Defect view after tumor removal. **f, g** Purse-string suture. **h** Complete defect closure.

Endoscopy_UCTN_Code_TTT_1AO_2AC

## Notes

Endoscopy E-Videos

https://eref.thieme.de/e-videos
*E-Videos* is an open access online section of the journal *Endoscopy* , reporting on interesting cases and new techniques in gastroenterological endoscopy. All papers include a high-quality video and are published with a Creative Commons CC-BY license. Endoscopy E-Videos qualify for HINARI discounts and waivers and eligibility is automatically checked during the submission process. We grant 100% waivers to articles whose corresponding authors are based in Group A countries and 50% waivers to those who are based in Group B countries as classified by Research4Life (see: https://www.research4life.org/access/eligibility/ ). This section has its own submission website at https://mc.manuscriptcentral.com/e-videos

## References

[1] Deprez P H Moons L MG OʼToole D Endoscopic management of subepithelial lesions including neuroendocrine neoplasms: European Society of Gastrointestinal Endoscopy (ESGE) Guideline Endoscopy 2022 54 412 429 35180797 10.1055/a-1751-5742

[2] Shichijo S Abe N Takeuchi H Endoscopic resection for gastric submucosal tumors: Japanese multicenter retrospective study Dig Endosc 2022 35 206 215 36165980 10.1111/den.14446

[3] Inoue T Shichijo S Nakajima K Novel protective retrieval device for a large rectal cancer specimen resected by endoscopic submucosal dissection Dig Endosc 2021 33 e129 e130 34245053 10.1111/den.14064

